# Community-, facility-, and individual-level outcomes of a district mental healthcare plan in a low-resource setting in Nepal: A population-based evaluation

**DOI:** 10.1371/journal.pmed.1002748

**Published:** 2019-02-14

**Authors:** Mark J. D. Jordans, Nagendra P. Luitel, Brandon A. Kohrt, Sujit D. Rathod, Emily C. Garman, Mary De Silva, Ivan H. Komproe, Vikram Patel, Crick Lund

**Affiliations:** 1 Centre for Global Mental Health, Health Service and Population Research Department, Institute of Psychiatry, Psychology and Neuroscience, King’s College London, London, United Kingdom; 2 Transcultural Psychosocial Organization Nepal, Kathmandu, Nepal; 3 Department of Psychiatry, George Washington University, Washington, District of Columbia, United States of America; 4 Department of Population Health, London School of Hygiene & Tropical Medicine, London, United Kingdom; 5 Alan J Flisher Centre for Public Mental Health, Department of Psychiatry and Mental Health, University of Cape Town, Cape Town, South Africa; 6 Wellcome Trust, London, United Kingdom; 7 Research and Development Department, Health-Works/HealthNetTPO, Amsterdam, The Netherlands; 8 Faculty of Social and Behavioural Sciences, Utrecht University, Utrecht, The Netherlands; 9 Department of Global Health and Social Medicine, Harvard Medical School, Boston, Massachusetts, United States of America; 10 Department of Global Health and Population, Harvard T.H. Chan School of Public Health, Boston, Massachusetts, United States of America; 11 Sangath, Goa, India; University of California, San Francisco, UNITED STATES

## Abstract

**Background:**

In low-income countries, care for people with mental, neurological, and substance use (MNS) disorders is largely absent, especially in rural settings. To increase treatment coverage, integration of mental health services into community and primary healthcare settings is recommended. While this strategy is being rolled out globally, rigorous evaluation of outcomes at each stage of the service delivery pathway from detection to treatment initiation to individual outcomes of care has been missing.

**Methods and findings:**

A combination of methods were employed to evaluate the impact of a district mental healthcare plan for depression, psychosis, alcohol use disorder (AUD), and epilepsy as part of the Programme for Improving Mental Health Care (PRIME) in Chitwan District, Nepal. We evaluated 4 components of the service delivery pathway: (1) contact coverage of primary care mental health services, evaluated through a community study (*N* = 3,482 combined for all waves of community surveys) and through service utilisation data (*N* = 727); (2) detection of mental illness among participants presenting in primary care facilities, evaluated through a facility study (*N* = 3,627 combined for all waves of facility surveys); (3) initiation of minimally adequate treatment after diagnosis, evaluated through the same facility study; and (4) treatment outcomes of patients receiving primary-care-based mental health services, evaluated through cohort studies (total *N* = 449 depression, *N* = 137; AUD, *N* = 175; psychosis, *N* = 95; epilepsy, *N* = 42). The lack of structured diagnostic assessments (instead of screening tools), the relatively small sample size for some study components, and the uncontrolled nature of the study are among the limitations to be noted. All data collection took place between 15 January 2013 and 15 February 2017. Contact coverage increased 7.5% for AUD (from 0% at baseline), 12.2% for depression (from 0%), 11.7% for epilepsy (from 1.3%), and 50.2% for psychosis (from 3.2%) when using service utilisation data over 12 months; community survey results did not reveal significant changes over time. Health worker detection of depression increased by 15.7% (from 8.9% to 24.6%) 6 months after training, and 10.3% (from 8.9% to 19.2%) 24 months after training; for AUD the increase was 58.9% (from 1.1% to 60.0%) and 11.0% (from 1.1% to 12.1%) for 6 months and 24 months, respectively. Provision of minimally adequate treatment subsequent to diagnosis for depression was 93.9% at 6 months and 66.7% at 24 months; for AUD these values were 95.1% and 75.0%, respectively. Changes in treatment outcomes demonstrated small to moderate effect sizes (9.7-point reduction [*d* = 0.34] in AUD symptoms, 6.4-point reduction [*d* = 0.43] in psychosis symptoms, 7.2-point reduction [*d* = 0.58] in depression symptoms) at 12 months post-treatment.

**Conclusions:**

These combined results make a promising case for the feasibility and impact of community- and primary-care-based services delivered through an integrated district mental healthcare plan in reducing the treatment gap and increasing effective coverage for MNS disorders. While the integrated mental healthcare approach does lead to apparent benefits in most of the outcome metrics, there are still significant areas that require further attention (e.g., no change in community-level contact coverage, attrition in AUD detection rates over time, and relatively low detection rates for depression).

## Abstract 2

Using data from Chitwan district in Nepal, Mark Jordans and colleagues reveal the benefits and impact of community-based mental health care in rural settings, where such care is often absent.

## Author summary

**Why was this study done?:**

- Following World Health Organization guidance on the integration of mental health into primary healthcare, there is a need for more evidence for the feasibility of the Mental Health Gap Action Programme (mhGAP) for scale-up of mental healthcare in low- and middle-income settings.
- This study evaluated a comprehensive mental healthcare plan at the district level in rural Nepal, a setting exemplifying scarce access to mental health services.

**What did the researchers do and find?:**

- After implementing a district mental health plan, the percentage of persons in the community receiving treatment increased from 0% to 12% for depression, 0% to 8% for alcohol use disorder, 3% to 53% for psychosis, and 1% to 13% for epilepsy.
- Six months after training, health workers detected 1 out of 4 patients with depression (1 out of 5 patients 2 years after training) and 3 out of 5 patients with alcohol use disorder (1 out of 8 patients 2 years after training) among patients presenting to primary care facilities. Of the patients detected with depression and with alcohol use disorder 95% received minimally adequate care (at 2 years after training this was 2 out of 3 for depression, and 3 out of 4 for alcohol use disorder).
- Patients treated for depression, alcohol use disorder, or psychosis showed small to moderate improvements in both symptoms and daily functioning at 12 months after starting treatment.

**What do these findings mean?:**

- Having health workers who are not specialists deliver community- and primary-healthcare-based mental health services is a promising strategy to increase the number of people with mental health problems benefiting from such care.
- Future implementation should increase supervision and quality assurance among the trained health workers, as well as ensuring that community-level interventions go hand-in-hand with those in health facilities.

## Introduction

Mental health is part of the Sustainable Development Goals, which set an agenda for improved treatment coverage by 2030 [1]. Treatment contact coverage is defined by the ratio of people who have contacted the service to the total target population in need of that service [2]. Increasing treatment coverage addresses the vast gap between availability of, and needs for, mental healthcare, especially in low- and middle-income countries (LMICs) [3,4]. The question is how to go about increasing coverage at a population level, especially in rural areas where there is little to no mental healthcare infrastructure. In keeping with the framework established by Tanahashi, which presents different levels of coverage related to the different stages of service provision [2], the fundamental issues underlying this question are (1) the allocation of resources in order to serve the maximum number of people, (2) the extent to which services are reaching the people they are intended for, and (3) the extent to which the services meet the people’s needs [2].

The integration of mental healthcare in community and primary healthcare settings has been advocated as a strategy to reduce the treatment gap in LMICs. The call for decentralised mental healthcare integrated into general health service settings has been made since the early 1970s, and this strategy was implemented through the WHO Collaborative Study on Strategies for Extending Mental Health Care [5]. Although there was limited success in implementing this strategy in LMICs during the following decades, renewed efforts have been made more recently. The World Health Organization (WHO) has developed the Mental Health Gap Action Programme (mhGAP) intervention guide, providing evidence-based clinical guidance for health workers to detect and diagnose mental illness [6]. Furthermore, recent reviews demonstrate promising results for psychological treatments by non-specialists in LMICs [7,8]. Task-sharing strategies are currently being adapted and implemented in many LMICs [9]. Yet, to date, there are few evaluations of coverage of mental health programmes [10], and to our knowledge none that combines evaluation methods at the community, facility, and individual levels to assess the impact of district mental healthcare plans (MHCPs). The aim of this report is to evaluate contact coverage, detection, and treatment outcomes as a result of a complex multi-component district-level mental healthcare programme for adults in Nepal.

## Methods

### Setting

The Programme for Improving Mental Health Care (PRIME) is a multi-country research programme that implements and evaluates district-level MHCPs in Ethiopia, India, Nepal, South Africa, and Uganda [11]. In Nepal, PRIME was implemented in Chitwan, a district in the south of the country with a total population of 579,984. During the evaluation phase the programme covered 10 primary healthcare facilities. Before the implementation of PRIME, mental health services were restricted to the district-level hospital. The Nepal health system consists of (1) district hospitals for specialised care, (2) primary healthcare centres for general medical care and first referral from health posts, and (3) the village-level health posts for basic health services. The major challenges in the existing health system ahead of implementing the MHCP were the lack of a formal government focal point for mental healthcare, the lack of basic psychotropic medicines in the essential medicines list, and the frequent transfer of primary health workers [12].

### Interventions

The MHCP that was developed and implemented in Nepal, in partnership with the Ministry of Health, has been described in detail elsewhere [13]. In summary, the MHCP comprised interventions at the community, health facility, and health service organisation levels—see Table 1. The community-level packages included community sensitisation, proactive case detection [14], and adherence support through home-based care. In addition, community counsellors were trained to provide the Healthy Activity Programme [15] for depression and Counselling for Alcohol Problems [16] for AUD. The facility-level packages included training and supervision for health workers to detect, diagnose, and initiate treatment (i.e., emotional support, psycho-education and psychotropic medication) for individuals with a diagnosis of a priority disorder (i.e., depression, psychosis, AUD, and epilepsy) following the mhGAP intervention guide [6]. In most LMICs, epilepsy is considered a psychiatric condition and is treated by mental health specialists. Because of this, the WHO mhGAP includes epilepsy in its priority mental health conditions in the intervention guidelines. Based on our priority-setting activity in Nepal [17], we determined that epilepsy should also be considered to be a priority mental health condition that could be treated in primary care settings. Finally, health-service-organisation-level packages included ensuring reliable supply of psychotropic medication, referrals to specialised care, and mechanisms for monitoring, capacity building, and resource mobilisation. For all services, regular ongoing supervision was part of the MHCP. Different types of service providers were involved in implementing the interventions. At the health facility, medical officers (5 to 6 years of training), health assistants (3 years of training), and auxiliary health workers (15 months of training) were involved in assessment, diagnosis, and management of priority mental health conditions. The staff nurse and auxiliary nurse mid-wife (18 months to 3 years of training) were responsible for providing brief psychosocial support in the health facilities. At the community level, counsellors are a new cadre of psychosocial workers trained by non-governmental organisations, responsible for providing psychological treatment to those referred by primary health workers. Female community health volunteers were responsible for proactive case detection and home-based care.

**Table 1:** Overview of the district mental healthcare plan.

Level	Interventions	Service provider	Training	Supervision
Health service organisation	Supervision of trained health workers	Psychiatrist	n/a	n/a
	Referral	Mental health professionals in district hospital	n/a	n/a
Health facility	Assessment, diagnosis, basic psychosocial support, and pharmacological treatment following mhGAP guidance	Primary health workers with authority to prescribe medication	9 days	Quarterly
	Focused psychosocial support	Primary health workers without authority to prescribe medication	5 days	
Community	Awareness raising about mental illness and availability of services	Community members at large, as well as specific groups such as mothers groups or traditional healers	1 day	n/a
	Proactive community case detection using the CIDT	Community health volunteers (i.e., FCHVs)	2 days	Monthly
	Focused psychosocial support consisting of HAP and CAP	Community counsellors	10 days	Bimonthly
	Home-based care to promote treatment adherence	Community health volunteers (i.e., FCHVs)	2 days	Monthly

Adapted from [13].

CAP, Counselling for Alcohol Problems; CIDT, Community Informant Detection Tool; FCHV, female community health volunteers; HAP, Healthy Activity Programme; mhGAP, Mental Health Gap Action Programme; n/a, not applicable.

### Study designs

**Fig 1:**
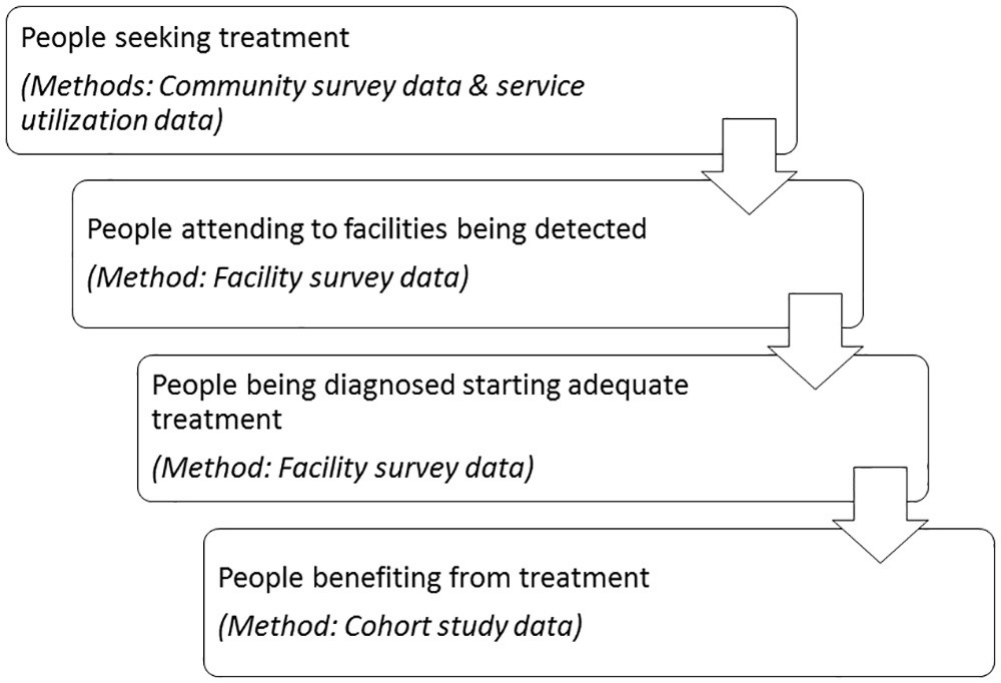
PRIME Nepal evaluation framework.

This paper presents the primary results of a collection of study designs, in order to present findings for each component in the process of evaluating the above-mentioned district MHCP: a community study, routine service utilisation data, a facility study, and cohort studies—described below (see Fig 1 and Table 2). The study designs and analysis plans have been described in detail elsewhere [13,18–21]; summaries are presented below.

**Table 2:** Overview of study components.

Component number	Research question	Method	Instruments	Sample size (*N*)
1	How many persons affected by depression, alcohol use disorder, psychoses, and epilepsy contact a health worker for help?	Community survey	PHQ-9 AUDIT	3,482
		Surveillance data	HMIS records	n/a
2	How many persons attending healthcare services are correctly detected with mental illness?	Facility survey	PHQ-9 AUDIT	3,627
3	How many persons diagnosed with MNS disorders receive minimally adequate care?	Facility survey	OPD records, reviewed by psychiatrist	
4	Are persons with MNS disorders benefiting from treatments delivered by primary health workers?	Cohort studies	PHQ-9 AUDIT WHODAS PANSS SIP-2R Epileptic seizures	449

AUDIT, Alcohol Use Disorders Identification Test; HMIS, health information management system; MNS, mental, neurological, and substance abuse; n/a, not applicable; OPD, outpatient department; PANSS, Positive and Negative Syndrome Scale; PHQ-9, Patient Health Questionnaire–9 item; SIP-2R, Short Inventory of Problems–Revised; WHODAS, WHO Disability Assessment Schedule.

We will structure the presentation of methods according to the 4 components of the service delivery pathway: (1) contact coverage of primary care mental health services, (2) detection of mental illness among participants presenting in primary care facilities, (3) initiation of minimally adequate treatment after diagnosis, and (4) the outcomes of patients receiving primary-care-based mental health treatment.

#### Evaluating changes in contact coverage

We conducted a community study to determine whether adults affected by depression or alcohol use disorder (AUD) were more likely to contact a health worker for help coinciding with the PRIME implementation period. A detailed description of the aims, design, recruitment, and questionnaire are available [19]. Briefly, 2 population-based cross-sectional surveys with independent samples were conducted, one before and one 30 months after implementation started. With 2,000 participants per round, the study had 80% power to detect a change in contact coverage from 5% to 25% among probable cases for each disorder, which we estimated would be 10% of the sample. Of the randomly selected adults (16 years and older, following Nepal legal classification) from randomly selected households in the implementation area, 99% provided informed written consent. The field workers orally administered a structured questionnaire that contained sections on demographic characteristics, food security, depression screening, depression symptoms in the past 12 months, and AUD screening. A probable case of depression had a PHQ-9 screening score of 10 or more or had depression-associated symptoms for at least 2 weeks in the past year [22]. A probable case of AUD had an AUDIT screening score of 9 or more [23]. Probable cases were asked whether they had contact with different health workers in the past 12 months, including non-specialist providers (e.g., medical officer, health assistant, auxiliary health worker) in government clinics. The timing of the data collection was as follows: baseline between May and July 2013 and endline between December 2016 and February 2017.

In addition, we used 1-year routine service utilisation data to assess change in contact coverage for all 4 priority disorders (depression, AUD, epilepsy, and psychosis). Change in contact coverage was calculated as the number of cases diagnosed with mental illness in 10 health facilities for a period of 12 months before the start of the MHCP and for 12 months during the implementation of the MHCP (baseline: 1 January–31 December 2013; endline: 25 August 2014–24 August 2015). The reasons for using both methods for assessing changes in contact coverage are that (1) service utilisation data were available for all 4 disorders, whereas the community survey only focused on depression and AUD, and (2) we were aware of the risk of being underpowered in the community survey, due to limited financial resources to conduct the survey.

#### Evaluation of changes in health workers’ detection of mental illness and initiation of adequate treatment

We conducted a facility study to determine whether adult attendees of primary healthcare facilities who were affected by depression or by AUD were more likely to be detected and adequately treated by clinicians during the PRIME implementation period [21]. Three cross-sectional surveys with independent sampling were conducted: before MHCP implementation and approximately 6 months and 24 months after initiating the MHCP. In the 10 health facilities, research staff recruited adults seeking outpatient services. All adult outpatients who were capable of providing informed written consent and who did not have an emergency medical problem were eligible for study recruitment. Among the eligible adult outpatients, 95% provided informed consent. In a private area adjacent to the waiting room, field workers verbally administered a structured questionnaire that contained sections on demographic characteristics and screening for depression and AUD. All participants who screened positive and a 10% random selection of screen-negative participants were given a consultation form for their clinician to complete and return to the participant. The form contained open-ended entries for diagnoses, treatments, advice, and referrals. A field worker made a copy of the form immediately after the consultation. A psychiatrist on the research team used the copy to determine whether each participant had been clinically diagnosed with depression or with AUD, and if so, whether there was evidence of minimally adequate treatment provision following mhGAP treatment guidelines. The timing of the data collection was as follows: baseline between September 2013 and February 2014, midline between August 2014 and August 2015, and endline between May and December 2016.

#### Evaluation of changes in treatment outcomes

We conducted 4 cohort studies to assess whether patients diagnosed with depression, AUD, psychosis, or epilepsy benefitted from receiving treatment under the MHCP. Patients were followed up for 1 year, to assess change in symptom severity and functional impairment, using a before-and-after comparison without control groups. A detailed overview of the methods has been previously published [20]. Briefly, individuals were eligible for inclusion in the treatment cohorts if they were diagnosed with 1 of the 4 priority conditions by a primary health worker in the health facilities implementing the MHCP. In addition, participants needed to be adults, living in the study district Chitwan, and willing to provide informed consent. For participants with psychosis, a caregiver was also recruited into the study to participate in a caregiver component of the study interview. Sample size was calculated based on a 20% reduction in symptom severity at the 12-month follow-up, with a 90% power and 2-sided alpha of 0.05, as well as an attrition rate of 15% to 20%. To allow the analysis of equity of treatment effects, the sample size was set at 200 for the depression and AUD cohorts, and at 150 for the psychosis and epilepsy cohorts. Patients were screened with the PHQ-9 and AUDIT by PRIME field workers before their consultation with the medical officer. They were then again followed up after their consultation to assess whether a diagnosis was made. If diagnosed, they were recruited into the respective treatment cohort. In case of patients with multiple diagnoses, priority was given to the more severe disorder. Participants were allocated to the psychosis or epilepsy cohort, in case of comorbidity with depression or AUD. If an individual was diagnosed with both AUD and depression, the participant was recruited into the AUD cohort. Baseline assessments were initiated at the clinic on the day of recruitment, and completed in the participants’ home, on average 1 day after recruitment. The follow-up assessments were conducted in the participants’ homes 3 months (depression and AUD) or 6 months after recruitment (psychosis and epilepsy), and again 12 months after recruitment (all cohorts). Data were collected using Android devices linked to an online application (Mobenzi, https://www.mobenzi.com). Participants were considered lost to follow-up if data for the 12-month assessment could not be collected. The timing of the data collection was as follows: baseline between September 2014 and August 2015, midline between December 2014 and November 2015, and endline between August 2015 and July 2016.

We mobilised the same field workers for all study components. Two months of extensive training was provided covering qualitative and quantitative research, interviewing skills, rapport building, informed consent, and inclusion/exclusion criteria. Additionally, 2 weeks of trainings were organised for each study component covering recruitment strategy and content of the questionnaire, including field practice. Field workers visited each sampled household or health facility, assessed eligibility criteria, performed the procedure for selecting participants, and obtained written consent among the selected participants for interviews. Interviews were conducted in a confidential place, and field workers used tablets for data collection.

### Study measures

For marital status, participants were grouped into 2 categories based on whether they had ever been married or not. All participants who were either working in an occupation sector or studying were put into a single ‘employed’ category. Participants were classified as being food insecure if anyone in their household had gone hungry in the past month due to lack of resources.

In the PHQ-9, participants reported the frequency with which they had experienced 9 symptoms over the past 2 weeks on a Likert scale ranging from 0 (‘not at all’) to 3 (‘nearly every day’), and scores from the 9 items were summed [24]. From a validation study in primary care settings in Nepal, a cutoff score of 10 or more had 94% sensitivity and 80% specificity, and internal consistency of α = 0.84 [22]. The 10-item AUDIT was developed by WHO and is used widely in LMICs [25]. With the sum of 10 items, a score of 8 or more is indicative of hazardous, harmful, or dependent drinking behaviours in the past year. Internal consistency of the AUDIT in Nepal has been shown to be α = 0.82 [23].

The 12-item interviewer-administered WHODAS 2.0 has items relating to difficulties engaging in daily activities due to health problems in the past 30 days, and items are scored on a Likert scale from 1 (‘none’) to 5 (‘extreme/cannot do’) [26]. The WHODAS has been validated in a range of settings [26], and has been used in previous research in Nepal [27]. Internal consistency for the WHODAS based on baseline data is α = 0.84 (depression cohort) and α = 0.85 (AUD cohort). Item response theory-based weights were used for the total scoring, to allow comparisons across populations. The assessment of accuracy of diagnosis and minimally adequate treatment for depression and AUD was done by a mhGAP-trained psychiatrist following predetermined decision rules based on the mhGAP intervention guide, stipulating inclusion and exclusion criteria for diagnosis and treatment (see S1 Table for criteria). In case of doubt, another psychiatrist (BAK) was consulted, to come to a consensus decision.

The 15-item SIP-2R is the short form of the Drinker Inventory of Consequences [28]. Each item is scored on a Likert scale from 0 (‘never’) to 3 (‘daily or almost daily’) with regard to the effects of drinking in the past 3 months. The 14-item PANSS is a symptom-based checklist for severity of psychosis symptoms [29]. The PANSS has not been validated in Nepal but has been culturally adapted for administration to patients and family members, with strong internal consistency for positive items (α = 0.82), negative items (α = 0.88), and combined (α = 0.89) in a rural sample in Nepal [30]. Internal consistency of the PANSS using the cohort baseline data was α = 0.84. The main outcome for patients with epilepsy was number of seizures in the past month. Participants in all studies were also assessed on a range of measures, including demographic and socio-economic, healthcare use, stigma, and discrimination measures (results not reported here).

### Statistical analyses

We collected data on demographic and health-related characteristics for participants who were recruited into the baseline rounds of the community, facility, and cohort studies. We summarised data using medians and interquartile ranges for continuous measures and counts and proportions for categorical measures.

For the community study participants with probable depression and with probable AUD, we reported the proportions who contacted any health worker or non-specialist health provider at each survey round. We used binomial regression to estimate the change in contact between rounds, and Cohen’s *h* for the effect size (ES). The regression estimates account for the complex survey design, i.e., strata and probability sampling weights. The analysis for participants with probable AUD was limited to men only, as previous analysis revealed that relatively few women had AUD [19]. These analyses were adjusted to account for the population-based survey design.

For calculating the change in contact coverage based on actual service utilisation data, we used the following equation:

$$Contact\;coverage=\frac{Number\;clinically\;diagnosed\;with\;a\;mental\;illness}{Prevalence\times Catchment\;population\;of\;health\;facilities}$$

The number of cases is based on all cases registered in health facility records over a period of 12 months. Baseline includes the total number of cases 12 months prior to PRIME, endline includes the total number of cases during 12 months when the PRIME cohort studies were implemented. The catchment population is the total adult population of the 10 Village Development Committees from the 2011 census (last available census data) (*N* = 63,189). Prevalence figures for depression and AUD are based on representative population-level prevalence rates from neighbouring India for (current) depression (2.7%), AUD (4.7%), and psychoses (0.4%) [31], and from a community study in Nepal for epilepsy (0.73%) [32].

For the facility study, for the participants who screened positive for depression or for AUD, we reported the proportions who returned their clinical consultation forms. Among those who returned their forms, we reported the proportions who had been diagnosed with depression or with AUD, and among those with a diagnosis, the proportions who had evidence of minimally adequate treatment provision. We used binomial regression to estimate the change in diagnosis at each round in comparison to the baseline round, and Cohen’s *h* for the ES. As it was not possible to use binomial regression to estimate the change in treatment at each follow-up round due to 0 counts in the baseline round, we used Fisher’s exact test to compare the proportions against the baseline round, and used a 1-sample test of proportions to calculate the 95% confidence intervals at each follow-up round.

For the cohort studies, differences in baseline demographic and clinical characteristics between participants with 12-month data and those lost to follow-up were assessed using non-parametric tests (Fisher’s exact test for categorical variables and Mann–Whitney U test for continuous variables). Because none of the continuous outcomes (WHODAS, PHQ-9, AUDIT, SIP-2R, and PANSS) were normally distributed, univariate negative binomial regression was used to assess change in total score on each outcome from baseline to midline and from baseline to endline in each cohort. Change in number of seizures in the past month in the epilepsy cohort was assessed using Poisson regression. Effect sizes (Cohen’s *d*) for paired sample analyses were calculated for each outcome. Equity of treatment effect by sex and caste was assessed using negative binomial regression, this time including sex or caste as an interaction term in the model. This was followed by a Wald chi-squared test.

All community, facility, and cohort data were analysed using Stata (StataCorp, College Station, Texas, US) version 14.

### Ethics

Ethical approval for the different study components was obtained from the Nepal Health Research Council; the Faculty of Health Sciences, University of Cape Town, South Africa; and the World Health Organization, Geneva, Switzerland.

## Results

### Change in contact coverage

In the baseline community survey round, 1,983 participants were screened, of whom 60% were male and 46% were between 30 and 50 years of age (see Table 3). Over 1 in 10 (11%) were probable cases of depression. Of the probable cases of depression identified at baseline, 8.5% had contacted a health worker in the past 12 months, in comparison to 11.8% of probable cases at endline; this change of +3.3% (95% CI −5.1%, 11.7%) was not significant. Contact with a non-specialist provider showed a non-significant increase of 2.3% (95% CI −2.8%, 7.5%). For probable cases of AUD among men, non-significant changes were observed for contact with any health worker (+6.3%, 95% CI −3.3%, 15.9%) and for contact with a non-specialist provider (+3.0%, 95% CI −1.6%, 7.6%) (Table 4). Based on actual service utilisation data over 12 months, we observed significant increases in contact coverage for all disorders. As shown in Table 5, the increases ranged from 7.5% for AUD to 50.2% for psychoses.

**Table 3:** Baseline demographic and clinical characteristics of the community surveys, facility surveys, and cohort samples.

Characteristic	Community survey (*N* = 1,983)		Facility survey (*N* = 1,252)		Depression cohort (*N* = 137)		AUD cohort (*N* = 175)		Psychosis cohort (*N* = 95)		Epilepsy cohort (*N* = 42)	
	*N* or median	Percent or SD	*N* or median	Percent or SD	*N* or median	Percent or IQR	*N* or median	Percent or IQR	*N* or median	Percent or IQR	*N* or median	Percent or IQR
**Sex**												
Male	1,280	60.1	813	64.9	19	13.9	149	85.1	50	52.6	25	59.5
Female	703	39.9	439	35.1	118	86.1	26	14.9	45	47.4	17	40.5
**Age**												
16–29	517	30.2	395	31.6	32	23.4	23	13.1	20	21.1	15	35.7
30–50	1,006	45.8	572	45.7	69	50.4	108	61.7	54	56.8	21	50.0
>50	460	24.0	285	22.8	36	26.3	44	25.1	21	22.1	6	14.3
**Marital status**												
No partner	215	86.4	114	9.1	26	29.0	10	5.7	30	31.6	15	35.7
Has a partner	1,768	13.6	1,138	90.9	111	81.0	165	94.3	65	68.4	27	64.3
**Religion**												
Hindu	1,604	80.4	1,066	85.1	118	86.1	134	76.6	79	83.2	33	78.6
Other	379	19.6	186	14.9	19	13.9	41	23.4	16	16.8	9	21.4
**Caste**												
Brahman/Chhetri (‘upper’ castes)	948	48.3	620	49.5	59	43.1	62	35.4	62	65.3	20	47.6
Janajati (ethnic minorities)	542	27.4	263	21.0	38	27.7	44	25.1	14	14.8	8	19.1
Dalit (‘lower’ castes)	308	15.0	291	23.2	32	23.4	50	28.6	9	9.5	10	23.8
Other	185	9.3	78	6.2	8	5.8	19	10.9	10	10.5	4	9.5
**Educational level**												
Uneducated/illiterate	275	13.2	196	15.6	38	27.7	37	21.1	22	23.2	10	23.8
Less than primary school	315	14.9	248	19.8	38	27.7	33	18.9	38	40.0	5	11.9
Primary school & above	1,393	72.0	808	64.5	61	44.5	105	60.0	35	36.8	27	64.3
**Employment**												
Not employed	87	4.7	35	2.8	92	71.3	37	21.6	64	68.8	26	61.9
Employed	1,896	95.3	1,217	97.2	37	28.7	134	78.4	29	31.2	16	38.1
**Food insecurity**												
No	14	0.9	352	96.7	7	5.4	5	2.9	90	3.2	0	0
Yes	455	99.1	12	3.3	122	94.6	166	97.1	3	96.8	42	100
**Outcome**												
PHQ-9 score	2.7	3.1	5.1	3.9	12.0	5.0	8.0	7.0	—	—	5.0	6.0
AUDIT score	1.4	3.5	2.0	4.7	0	1.0	27.0	9.0	—	—	0	0
WHODAS score	16.1	3.5	18.2	5.9	36.1	25.0	19.4	22.2	27.8	27.8	19.4	22.2
SIP-2R	—	—	—	—	—	—	20.0	13.0	—	—	—	—
PANSS score	—	—	—	—	—	—	—	—	11.0	12.0	—	—
Number of seizures	—	—	—	—	—	—	—	—	—	—	1.0	3.0

AUD, alcohol use disorder; AUDIT, Alcohol Use Disorders Identification Test; IQR, interquartile range; PANSS, Positive and Negative Syndrome Scale; PHQ-9, Patient Health Questionnaire–9 item; SIP-2R, Short Inventory of Problems–Revised; WHODAS, WHO Disability Assessment Schedule.

**Table 4:** Contact with a health worker for depression or for alcohol use disorder among adult residents based on 12-month recall, Chitwan District, Nepal, 2013–2017.

Outcome	Baseline *N* = 1,983	Endline *N* = 1,499	Percent difference baseline to endline (95% CI)
**Depression**			
Probable case (%)	228/1,983 (11.0)	118/1,499 (7.6)	
Any health services contact—total (%)	18/228 (8.5)	13/118 (11.8)	+3.3 (−5.1, 11.7)
Primary care contact—total (%)	5/228 (1.8)	4/118 (4.2)	+2.3 (−2.8, 7.5)
**Alcohol use disorder (male only)**	*N* = 703	*N* = 427	
Probable case (%)	89/703 (12.0)	66/427 (13.8)	
Any health services contact—total (%)	5/89 (5.4)	9/66 (11.7)	+6.3 (−3.3, 15.9)
Primary care contact—total (%)	1/89 (0.6)	3/66 (3.7)	+3.0 (−1.6, 7.6)

Counts are presented as observed, while percentages are design-adjusted; the percent difference is change in treatment contact, calculated with design-adjusted binomial regression models, estimated with binomial regression models that account for the complex survey design, i.e., strata and probability sampling weights.

**Table 5:** Contact coverage rates for people with probable depression, AUD, psychosis, and epilepsy using 12-month service utilisation data.

Disorder	Contact coverage rate	
	Baseline (%)	Endline (%)
Depression	0	12.2
AUD	0	7.5
Psychosis	3.2	53.4
Epilepsy	1.3	13.0

AUD, alcohol use disorder.

### Detection of persons with mental illness

In the baseline round, 1,252 participants were screened, of whom 65% were male and 46% were between 30 and 50 years of age (Table 3). There were 186 participants (15%) who screened positive for depression, of whom 179 returned their outpatient consultation forms. Using outpatient form data, 16/179 (8.9%) were judged to have received a diagnosis of depression. The proportion receiving a diagnosis increased from baseline by 15.7% (95% CI 7.3%, 24.0%), with an ES of 0.432, at the midline round and by 10.2% (95% CI 1.2%, 19.2%; ES 0.301) at the endline round. There were 92 participants (7.4%) who screened positive for AUD. Diagnosis increased from baseline by 58.9% (95% CI 42.0%, 75.7%; ES 1.562) at midline and by 11.0% (95% CI 0.7%, 21.3%; ES 0.500) at endline (Table 6).

**Table 6:** Clinical detection of depression and of AUD among adult government health clinic attendees, Chitwan, Nepal, 2013–2017.

Disorder	Baseline (BL) (*N* = 1,252)	Midline (ML) (*N* = 1,396)	Endline (EL) (*N* = 979)	ML minus BL (95% CI) or ML (95% CI)	Cohen’s *h* for ML minus BL	EL minus BL (95% CI) or EL (95% CI)	Cohen’s *h* for EL minus BL
**Depression**							
Screen positive (%)	186/1,252 (14.9)	143/1,396 (10.2)	99/979 (10.1)				
Screen positive with consultation data (%)	179/186 (96.2)	134/143 (93.7)	94/99 (95.0)				
Detected (%)	16/179 (8.9)	33/134 (24.6)	18/94 (19.2)	+15.7* (7.3, 24.0)	0.432	+10.2* (1.2, 19.2)	0.301
Adequate treatment	0/16 (0.0)	31/33 (93.9)	12/18 (66.7)	93.9** (77.9, 98.6)		66.7** (41.7, 84.8)	
**AUD—men only**							
Screen positive (%)	92/1,252 (7.4)	180/1,396 (12.9)	38/979 (3.9)				
Screen positive with consultation data (%)	90/92 (97.8)	170/180 (94.4)	33/38 (86.8)				
Detected (%)	1/90 (1.1)	102/170 (60.0)	4/33 (12.1)	+58.9* (42.0, 75.7)	1.562	+11.0* (0.7, 21.3)	0.500
Adequate treatment	0/1 (0.0)	97/102 (95.1)	3/4 (75.0)	95.1** (88.6, 98.0)		75.0** (17.6, 97.7)	

Detection proportion differences and *p*-values estimated using binomial regression accounting for clinic-level clustering, and standard errors adjusted for clinic-level clustering.

*p < 0.05 for difference of coverage from baseline round using binomial regression.

**p < 0.05 for association of coverage with baseline round, using Fisher’s exact test.

AUD, alcohol use disorder.

### Initiation of adequate treatment

Among facility survey participants who received a depression diagnosis at baseline, none received adequate treatment. At midline, among those diagnosed, 93.9% (95% CI 77.9%, 98.6%) received adequate treatment, as did 66.7% (95% CI 41.7%, 84.8%) at endline. Among those diagnosed with AUD, 95.1% (95% CI 88.6%, 98.0%) had adequate treatment at midline and 75.0% (95% CI 17.6%, 97.7%) at endline, up from 0% at baseline (see Table 6).

### Clinical and functional treatment outcomes

A total of 2,139 patients were eligible and consented to take part in the cohort studies. Of these, 137 received a primary diagnosis of depression, 175 were diagnosed with AUD, and 42 were diagnosed with epilepsy—all were recruited into the respective cohort. A total of 95 caregivers of patients diagnosed with psychosis were also recruited into the psychosis cohort. Participants’ demographic characteristics are presented in Table 3. Attrition at the 12-month follow-up was 20.0%, 18.9%, 9.5%, and 9.5% for the depression, AUD, psychosis, and epilepsy cohorts, respectively. Participants lost to follow-up differed from active participants only in the epilepsy cohort: they were all single, and had greater baseline WHODAS and PHQ-9 scores (see S2 Table for reasons for loss to follow-up).

Results of the negative binomial regressions and Poisson regression are presented in Table 7. Participants in the depression cohort showed significant improvement from baseline to endline, with a significant reduction in WHODAS (β = −15.89; 95% CI −23.03, −8.74; *d* = −0.41) and PHQ-9 scores (β = −7.22; 95% CI −9.54, −4.89; *d* = −0.58). In addition, 68.2% (95% CI 58.8%, 76.3%) of participants showed a 50% reduction in PHQ-9 (response) at endline. In the AUD cohort, change in score from baseline to endline was significant for the WHODAS (β = −8.57; 95% CI −12.64, −4.49; *d* = −0.35), AUDIT (β = −9.68; 95% CI −14.35, −5.00; *d* = −0.34), and SIP-2R (β = −9.13; 95% CI −12.73, −5.54; *d* = −0.42). Change in WHODAS score from baseline to endline among the psychosis cohort was also significant (β = −13.56; 95% CI −20.78, −6.34; *d* = −0.40), and so was change in PANSS score (β = −6.42; 95% CI −9.55, −3.28; *d* = −0.43). Change in WHODAS or symptom severity score was also significant at midline in the depression, AUD, and psychosis cohorts. However, change in WHODAS score or number of seizures in the epilepsy cohort was not significant, neither at midline nor endline. Moreover, among participants who scored above the validated PHQ-9 cutoff for depression at baseline, 86.7% (95% CI 77.8%, 92.3%) of the depression cohort scored below the cutoff at endline. For AUD, 31.9% (95% CI 24.7%, 40.1%) of participants scoring above the validated AUDIT cutoff at baseline scored below cutoff at endline.

**Table 7:** Impact of PRIME mental healthcare plan on individual-level outcomes.

	Midline					Endline				
	*N*	Mean (SD) or *N* (%)	Mean change from BL (β) or RR	95% CI	Cohen’s *d*	*N*	Mean (SD) or *N* (%)	Mean change from BL (β) or RR	95% CI	Cohen’s *d*
**Depression**										
PHQ-9 score	110	4.7 (4.32)	−7.32***	−9.64 to −5.00	−0.59	111	4.8 (4.60)	−7.22***	−9.54 to −4.89	−0.58
WHODAS	110	18.9 (18.83)	−16.77***	−23.83 to −9.70	−0.44	111	19.8 (20.92)	−15.89***	−23.03 to −8.74	−0.41
**AUD**										
AUDIT score	135	10.4 (9.87)	−15.00***	−19.26 to −10.75	−0.59	142	15.7 (10.74)	−9.68***	−14.35 to −5.00	−0.34
SIP-2R score	135	8.5 (9.30)	−11.50***	−14.93 to −8.08	−0.57	142	10.9 (9.52)	−9.13***	−12.73 to −5.54	−0.42
WHODAS score	135	12.3 (15.77)	−9.84***	−13.83 to −5.85	−0.42	142	13.6 (14.33)	−8.57***	−12.64 to −4.49	−0.35
**Psychosis**										
PANSS score	87	6.7 (6.84)	−6.30 ***	−9.44 to −3.15	−0.42	86	6.6 (7.72)	−6.42***	−9.55 to −3.28	−0.43
WHODAS score	87	21.0 (18.45)	−9.19*	−16.90 to −1.47	−0.25	86	16.7 (18.67)	−13.56***	−20.78 to −6.34	−0.40
**Epilepsy**										
Number of seizures	40	3.9 (10.02)	0.35	0.29 to 0.42		38	2.7 (5.60)	0.24	0.29 to 0.42	
WHODAS score	40	16.0 (18.47)	−3.14	−10.99 to 4.71	−0.12	38	14.5 (15.95)	−4.71	−12.32 to 2.91	−0.20

*p < 0.05;

***p < 0.001.

AUDIT, Alcohol Use Disorders Identification Test; BL, baseline; PANSS, Positive and Negative Syndrome Scale; PHQ-9, Patient Health Questionnaire–9 item; PRIME, Programme for Improving Mental Health Care; RR, risk ratio; SD, standard deviation; SIP-2R, Short Inventory of Problems–Revised; WHODAS, WHO Disability Assessment Schedule.

Equity analyses suggest that change in the primary outcomes for the depression cohort (PHQ-9), AUD cohort (AUDIT and SIP-2R), and psychosis cohort (PANSS) did not differ according to the sex or caste of the participants. In the epilepsy cohort, however, the decrease in number of seizures in the past month from baseline to endline was significantly greater among men compared to women (χ^2^ = 10.4, *p* < 0.001). The decrease in number of seizures from baseline to endline was also greater among the ‘upper’ caste groups (Brahman/Chhetri) (χ^2^ = 47.35, *p* < 0.001). This was due to 1 outlier (>100 seizures reported by a participant of the Brahman caste). When outliers were excluded, change in number of seizures over time was no longer different by sex, but was significantly lower from baseline to midline among the ‘lower’ and ethnic minority castes (Janajati, Dalit, or other) (χ^2^ = 61.4, *p* < 0.001).

## Discussion

These combined outcomes demonstrate promising results of a district-level MHCP in a low-resource community and primary care setting. We see improvements in actual contact coverage, detection of mental illness by trained health workers, the initiation of minimally adequate treatment, and treatment outcomes. Together these results show the potential of a district MHCP to increase effective coverage for MNS disorders. However, there are also important areas that require further attention, such as preventing attrition in AUD detection rates over time, improving detection rates for depression, maintaining adequacy of treatment over time, and achieving better treatment outcomes for some disorders.

This research programme is, to our knowledge, unique in that it aims to evaluate each of the steps in the process of integrating mental healthcare in community and primary healthcare platforms in a low-income setting. Through a combination of studies, it provides a population-level perspective on the impact of a district-wide MHCP, covering the extent to which (1) people are seeking care at health facilities, (2) disorders in people attending health facilities are being detected, (3) people being diagnosed are starting adequate treatment, and (4) people are benefiting from treatment.

### People seeking treatment (contact coverage)

Based on a representative community study, we see modest, non-significant increases in contact coverage as a result of introducing the district-level MHCP; our measure included any treatment contact and contact with a primary health worker. The endline rate of 4%, however, does not come close to the targets that were set at the onset of the programme [33]. One possible explanation for this is that the community surveys, although representative, were underpowered to detect changes at the population level. True coverage change may be estimated more efficiently by combining routine clinic data with population prevalence estimates from national surveys [10]. For example, the change in contact coverage using actual service utilisation data is especially promising for psychosis, for which we achieved the target of 50% coverage. In interpreting the contact coverage rates using routine clinic data, it is important to keep in mind that these are based on contact with primary healthcare services. Contact with specialised services is excluded from the calculation and may explain why the baseline rates are low, especially for epilepsy and psychosis, compared to coverage rates in other studies and settings.

When interpreting the different estimates of contact coverage, it is important to note that routine clinic data provide a more accurate measure of the numbers actually taking up services while the community survey is a more accurate measure of the proportion of the population at large seeking treatment. The former is limited in that it is difficult to ascertain the characteristics of people who need but do not seek care, and the latter is limited by underestimating numbers who actually take up care or by problems of requiring large samples in order to be adequately powered.

The changed rates in treatment coverage reported in this study are similar to rates seen in high-income settings [3]. One of the elements that may have contributed to increased service utilisation, besides availability of services, is the proactive community case detection strategy that was part of the approach. Utilisation of the CIDT has been demonstrated to be a viable strategy to increase help-seeking for mental healthcare [34,35]. In future programmes the use of the CIDT should be combined with effective stigma-reduction interventions within the communities [36], in order to combine supply-side strategies with strategies that increase demand for mental healthcare.

### People with disorders being detected when attending facilities

Once accessing health facilities with supervised mhGAP trained health workers, 3 out of 5 people with alcohol problems and 1 out of 4 with depression are detected when the knowledge and skills from training are still relatively fresh (6 months after training). Despite the detection rate remaining relatively stable for depression (1 out 5 patients at 2 years post-training), we see a big drop for AUD (1 out of 8 patients at 2 years), which is possibly due to high dropout rates over time, resulting in health workers losing faith in treating AUD. Although we see significant increase in detection of depression and AUD, many people with depression complaints still go undetected. This is not entirely unsurprising given the difficulties in diagnosing depression in primary care settings, also in high-income settings [37]. The global gaps in primary health workers’ detection of depression require further examination and potentially the development of new training and supervision strategies. One suggestion is to reframe the diagnosis of depression in primary care not as a binary approach, but as a staging approach to the identification and classification of mental disorder [38]. Finally, in our study the small change in depression is influenced by the high baseline detection rate for depression (nearly 9%), which is likely attributable to 1 health worker who had received mental health training in another location.

With a cutoff score of 10 in primary care settings in Nepal, the PHQ-9 misclassifies approximately 6 participants as false positives for every 4 participants who are true positives [22]. This is comparable to false positive rates with the PHQ-9 in high-income settings. With this in mind, the identified detection rates at baseline, midline, and endline using the PHQ-9 likely underestimate the true detection rate given the high number of PHQ-9 false positives. Working with a PHQ-9 false detection rate of 60% and assuming that the primary health workers only identified true positives, then the upper limit for accurate detection of depression may have been 22% at baseline, 60% at midline, and 50% at endline. The actual detection rate likely falls somewhere below these rates and above the PHQ-9 results reported in our results section. Future studies should consider using structured diagnostic questions for confirmation of detection rates with primary health workers.

### People being diagnosed starting adequate treatment

Nearly all (95%) people that health workers correctly detected with depression or AUD received minimally adequate treatment 6 months post-training. Twenty-four months after the training, we see a decline to 2/3 for depression and 3/4 for AUD. Importantly, these high rates support the feasibility of relatively short and focused training of health workers, which is at the heart of WHO’s mhGAP programme [39]. These results also re-emphasise the need for supervision to keep up good practice over time [40,41]. At the same time, it is worth noting that coding of treatment adequacy was based on ‘minimally adequate’ care. Unstable supply of psychotropic medicines during the early phase of the programme may explain some of the decline in adequate treatment. Further research is needed assessing the rate of optimal care (this study is currently ongoing, and will be published separately).

### People benefiting from treatment

The combination of interventions provided through the MHCP—which includes psychotropic treatment, home-based care, and psychological treatments—has the expected beneficial effects for people with depression, AUD, and psychosis. At 12 months after treatment initiation, all cohorts see an 8%–16% reduction of functional impairment and 6%–10% symptom reduction (at midline, these values are 6%–15% and 9%–17%, respectively). For people with depression, 87% score below the cutoff of the validated symptoms checklist at 12 months post-treatment; for people with AUD this value is 32%. This study did not aim to evaluate the effectiveness of the provided treatments per se. Rather, it aimed to assess improved functioning and symptom reduction as indicators of feasibility of a community MHCP provided by non-specialists. Our findings support this feasibility, with the exception of the epilepsy cohort, which did not see significant improvement. That said, not having a control group remains an important limitation, especially given trends towards natural remission among people with depression and AUD [42]. The improvements among people with psychosis are similar to those in another recent study of mhGAP in a different rural region of Nepal [30]. Improvements among depressed patients are especially driven by the added value of psychological treatment by the community counsellors, whereas for patients with AUD, pharmacological treatment and psychoeducation by primary health workers appear to primarily explain the improvement [43]. The overall absence of treatment effects for epilepsy is surprising given established effectiveness of treatment as included in the mhGAP guidelines, as well as positive prior outcomes in Nepal [30]. There are a few possible explanations for this finding: (1) a relatively small sample size might have made for an underpowered study and (2) 40% of participants did not report any seizures in the month before baseline. The reasons for the lower change in number of seizures among the ‘lower’ castes and ethnic minority groups need to be studied further.

The strength of this study is that it presents an evaluation of a real-world district-wide implementation of mental health services within community and primary healthcare platforms in a low-income country. The evaluation follows a theory of change that was developed at the outset of the programme [41], based on guidelines for the evaluation of complex interventions [44], and coordinated with studies in 4 other LMICs [18].

There are several limitations to be noted. First, the use of screening tools (e.g., PHQ-9 and AUDIT) rather than structured diagnostic assessment risks misclassification of cases in the community and facility studies and an associated reduction of statistical power. Second, community- and facility-level impacts of PRIME for epilepsy and psychosis remain unknown, as these were not included in the study components. Third, while the assessment of adequacy of initiated treatment was done by a mhGAP trained psychiatrist using predefined criteria, we did not systematically assess the reliability of that assessment. Fourth, as noted above, the community surveys might have been underpowered. The sample size suggested by power analysis was reduced for budgetary reasons. We compensated for this by also evaluating changes in contact coverage using service utilisation data. Fifth, the study designs are observational and uncontrolled, which increases the risk for biases.

This study has several implications for future implementation and research into scaling up mental healthcare in LMICs. First, for any population-level programme, it is essential to demonstrate changes in contact coverage. A programme like PRIME appears to improve contact coverage based on 12-month service utilisation data while failing to demonstrate such change using a representative community survey. Future studies evaluating contact coverage using representative samples might need to work with larger samples. Although over half of the people with psychosis appear to have been reached by the programme, there should be more focus on getting people into care, especially for depression, epilepsy, and AUD. Second, and related to the above, investments in making services available should be combined with efforts to increase demand for these services, for example by using proactive case-finding tools such as the CIDT [34]. A combined demand- and supply-side approach will optimise uptake and utilisation of care. Third, a brief mhGAP training to health workers appears adequate for drastically improving their capacity to detect cases of, and initiate minimally adequate treatment for, depression and AUD. At the same time, the attrition of detection rates for AUD over time calls for more focus on supervision and quality monitoring, and the depression detection rates still leave room for improvement, possibly by using different approaches to diagnosis. Fourth, while individuals with depression, AUD, and psychosis receiving mhGAP-based pharmacological and psychological treatment from non-specialist providers report clinical improvements, most of the changes have relatively small ESs, which calls for more focus on the quality of care in future implementation as a means of boosting clinical outcomes. Given the lack control groups, it is not possible to account for natural remission of symptoms as an explanation for these changes. Similarly, the lack of improvements within the overall epilepsy cohort requires further investigation. Taken together, these findings show encouraging improvements in effective coverage at the population level following the implementation of a local MHCP.

### Conclusion

In efforts to respond to the enormous treatment gap for people with mental illness in LMICs, there is an urgent need for evidence regarding the feasibility of scaling up mental healthcare through community and primary healthcare platforms. PRIME is, to the best of our knowledge, the first programme to systematically evaluate the different assumptions about, and steps towards, making effective mental healthcare available at a population level. A primary indicator of success is effective coverage, defined as the proportion of people who need treatment who accessed services resulting in improvements in patient clinical and functional outcomes. Combining the results from the community, facility, and cohort studies, the programme appears to achieve effective coverage of 1 out of 34 participants with depression and 1 out of 23 participants with AUD—based on community and primary care services alone. Another important indicator is the extent of change that is the result of the implementation of the district MHCP. We demonstrated modest to large and targeted changes in contact coverage (ranging from 1 out of 13 participants with AUD to half of all patients with psychosis). Changes in health workers’ detection ranged from a small ES for change in health worker detection of depression at 24 months (*d* = 0.30) to a large ES for change in detection of AUD post-training (*d* = 1.6). We demonstrated that minimally adequate treatment was initiated at the lowest level for two-thirds of the cases with depression at endline, and up to 95% of the cases with AUD right after training. Finally, 3 months after patients initiated care, we observed small to moderate ESs for clinical outcomes (ranging from *d* = 0.25 for improved functioning among people with psychosis to *d* = 0.59 for reduction in symptoms for depression and AUD), changes that are maintained 12 months after starting treatment.

These combined results make a strong case for the impact of a district MHCP in reducing the treatment gap and increasing effective coverage for priority mental disorders, while also pointing towards a set of strategies and new research questions that can contribute towards additional improvements for the future. Ultimately, populations in other low-income and fragile states with limited or non-existent mental health services desperately need models that build on the lessons learned in Nepal through PRIME’s public mental healthcare model.

## Supporting information

S1 STROBE Statement for cohort studies. (DOC)

S1 Table Decision criteria for minimally adequate treatment. (DOCX)

S2 Table Reasons for loss to follow-up in the cohort studies. (DOCX)

## Abbreviations

AUD: alcohol use disorder
AUDIT: Alcohol Use Disorders Identification Test
CIDT: Community Informant Detection Tool
ES: effect size
LMICs: low- and middle-income countries
mhGAP: Mental Health Gap Action Programme
MHCP: mental healthcare plan
MNS: mental, neurological, and substance abuse
PANSS: Positive and Negative Syndrome Scale
PHQ-9: Patient Health Questionnaire–9 item
PRIME: Programme for Improving Mental Health Care
SIP-2R: Short Inventory of Problems–Revised
WHO: World Health Organization
WHODAS: World Health Organization Disability Assessment Schedule
