# Supplementary material for: Community-, facility-, and individual-level outcomes of a district mental healthcare plan in a low-resource setting in Nepal: A population-based evaluation
Source: PLoS Med. 2019 Feb 14;16(2):e1002748. doi: 10.1371/journal.pmed.1002748 (PMC6375569; doi:10.1371/journal.pmed.1002748)
Supplement: S1 Table — (DOCX) [file pmed.1002748.s002.docx]

Supplementary Table 1: Criteria for determining minimally adequate care

| Detection of depression | Treatment of depression |
| --- | --- |
| *Included:*   - Diagnosis of depression - Diagnosis of anxiety stress - Diagnosis of bipolar   *Excluded:*   - Diagnosis of insomnia, tension headache, stress, headache, behavioral problem, mental disorder, psychiatric problem   schizophrenia, epilepsy, bipolar, central nervous system problem | *Included:*   - Prescription of fluoxetine, amitriptyline or other antidepressant, regardless of dosage - Referral to a mental health specialist - “Advice on stress” and “education” included only when there is a depression diagnosis - Amitriptyline prescription included only when there is a diagnosis - Counselling or talking treatment included only when there is a diagnosis - Providing advice (psycho-education) on coping strategies for depression (e.g. activating, healthy life-style) - Healthy Activity Program (HAP)   *Excluded:*   - Diazepam without an anti-depressant (SSRI or TCA) - Antipsychotic without an anti-depressant (SSRI or TCA) - Non-specific referrals (e.g. "hospital") |
| Detection of AUD | Treatment of AUD |
| *Included:*   - Diagnosis of AUD or drinking problem   *Excluded:*   - Drug abuse or other substance use problems | *Included:*   - Referral to a mental health or addictions specialist - Diazepam included only when there is a diagnosis AND thiamin/Vitamin B is given. - Vitamin B included only when there is a diagnosis - Counselling or talking treatment included only when there is a diagnosis - Providing advice (psycho-education) on coping strategies/ motivational interviewing for AUD (e.g. reducing intake, preventing situations that lead to intake) - Counseling for Alcohol Problems (CAP)   *Excluded:*   - Non-specific referrals (e.g. "hospital") - Only diazepam |
