# Supplementary material for: Community-, facility-, and individual-level outcomes of a district mental healthcare plan in a low-resource setting in Nepal: A population-based evaluation
Source: PLoS Med. 2019 Feb 14;16(2):e1002748. doi: 10.1371/journal.pmed.1002748 (PMC6375569; doi:10.1371/journal.pmed.1002748)
Supplement: S2 Table — (DOCX) [file pmed.1002748.s003.docx]

Supplementary Table S2. Reasons for loss to follow-up in the cohort studies

| **Reason** | **Depression** | **AUD** | **Psychosis** | **Epilepsy** |
| --- | --- | --- | --- | --- |
| Moved away | 13 | 18 | 2 | 0 |
| Refused | 11 | 6 | 2 | 2 |
| Not at home after several visits | 0 | 3 | 1 | 1 |
| Kept cancelling | 1 | 1 | 0 | 0 |
| Hospitalisation | 1 | 0 | 0 | 0 |
| Death | 0 | 2 | 4 | 1 |
| Other | 0 | 3 | 0 | 0 |
| Total | 26 | 33 | 9 | 3 |

*Note.* AUD = Alcohol Use Disorder
